# Green practices and sustainability as precursors to the intention to participate again in sporting events held in nature

**DOI:** 10.3389/fspor.2025.1541485

**Published:** 2025-03-06

**Authors:** Karla Chaves-Castro, Guillermo Morán-Gámez, Alberto Nuviala, Antonio Fernández-Martínez

**Affiliations:** ^1^ School of Physical Education and Sports, University of Costa Rica, San José, Costa Rica; ^2^ Faculty of Education Sciences, University of Cádiz, Cádiz, Spain; ^3^ Department of Physical Education and Sport, Faculty of Sports Sciences, University of Pablo de Olavide, Seville, Spain

**Keywords:** sustainability, green practices, quality, satisfaction, emotions, future intentions

## Abstract

**Introduction:**

In recent years, the need to tackle major environmental challenges has increased awareness among organisations seeking to promote sustainable practices and minimise the impact of their events. This is a challenge for sports organisations and promoters, as they must offer high-quality events that meet the needs of the participants and, at the same time, are environmentally friendly.

**Objective:**

To explore the relationship between sustainability, green practices, quality, satisfaction and intentions to participate again in sports events held in nature in Costa Rica. Methodology: 781 participants (66.3% men and 33.7% women), with an average age of 40.9 ± 11.9 years. We used a questionnaire composed of 24 items taken from different validated scales. For the purpose of data analysis, we applied various statistical techniques, including the calculation of descriptive statistics, Cronbach's alpha index, the average variance extracted, composite reliability and structural equation models.

**Results:**

Sustainability is an important determining factor of event quality. We also found that green practices positively influence event quality. In addition, the emotions experienced by the participants have a significant impact on event quality, as well as on their intentions to participate again in the future.

**Conclusions:**

The implementation of green and sustainable practices is fundamental in the management of sporting events held in natural spaces, since they not only contribute to reducing environmental impact, but can also improve the quality of the event. In turn, these variables, along with the positive emotions experienced during the event, influence the participants' intentions to participate again on future occasions.

## 1 Introduction

In recent years, the need to tackle the major social and environmental challenges we are facing has led business organisations to develop corporate sustainability practices within their possibilities, which are not limited exclusively to environmental issues but which, according to Ghobakhloo (1), and with the aim to being competitive, encompass everything related to the preservation of the economy and natural resources, as well as the problems of inequality, peace, injustice. All of this affects both their strategy and performance, even impacting customers (2019).

The sports industry has not been left out of this trend because, given the current climate, Casper et al. (2) argue the need to strike a balance between the economy and the environment. The motivations of sports organisations to implement these strategies do not only respond to concern for the environment, but also arise from processes of organisational legitimacy, the need to set themselves apart from the competition and to adapt to current demands (3). There is evidence that sports organisations often engage in the implementation of sustainable strategies, such as green practices, in response to political, social and functional pressures (4), and even, according to Todaro et al. (5), in relation to the potential economic and image benefits that can be obtained. Ulloa-Hernández et al. (6) classify the state of research related to sport and sustainability as being in its infancy, with a long way to go in terms of innovation and research (7).

Despite the relevance of the topic, the scientific literature related to the performance of sports organisations has mainly focused on studying aspects associated with perceived service provision, such as quality, satisfaction, and how these relate to intentions to participate again in the future, which provides organisations with key information to improve their activities (8, 9). The literature indicates that the quality of an event is related to satisfaction and that it also influences participants' intentions to participate again in the future (10). The number of studies conducted in the sports industry, in terms of services or events, has increased in recent years (11, 12), having ignored personal aspects such as emotions experienced during the use of the service, as these could influence perceived event quality and satisfaction (13–15).

Another line of work has shown that the inclusion of sustainability and green practices in the management of sporting events improves internal processes, enhances organisational image and optimises service provision (4, 5), aspects that are associated with perceived quality and user satisfaction. Despite the relevance of the topic and the possible relationship between these variables, no previous studies have linked them. Therefore, the aim of this work is to explore the relationship between sustainability, green practices, quality, emotions, satisfaction and the intention to participate in future sporting events held in nature. This would allow us to understand how sustainability and/or green practices, as strategies used by organisers of services and/or sporting events, affect important variables such as quality, emotions and satisfaction, which condition the future behaviours of users of these services.

## 2 Conceptual framework and hypothesis

### 2.1 Sustainability in the sports environment

Sustainability as a concept arises from scientific debates regarding the limits of economic growth, pressures on environmental systems and the role of human beings in nature (16). It is a term that responds to the imbalance generated between the environment and the economy, resulting from the lifestyle of industrialised nations and their excessive development during the sixties and seventies (17). Some authors define sustainability as a way to meet the needs of people today without jeopardising the possibilities of future generations also being able to do so (18, 19).

Thus, sustainability refers not only to matters related to natural resources or the environment, but also includes economic and social aspects (16, 20). In the literature, the Triple Bottom Line theory is used to explain the growing interest of sports organisations to be more responsible towards the environment, being able to obtain good results in all three areas (21, 22). The environmental initiatives framed within this theory refer to actions that minimise the environmental impact of the organisation; those of a social nature reside in the capacity that sports organisations have to influence the opinions and behaviours of users; and economic initiatives usually come in the form of cost savings through the application of environmental sustainability initiatives. Sports organisations that adopt these measures can enjoy direct and indirect economic benefits, such as lower costs and more positive perceptions among the general public (23, 24).

A sporting event is considered sustainable when it seeks to minimise the level of environmental impact in the place where it is held, while also seeking progressive social and economic development for the areas where it takes place (20). In addition, a sustainable sporting event should offer people options for accessibility and integration, promote the responsible use and consumption of natural resources and leave a positive legacy linked to the practice of sustainable habits (22).

Despite the fact that sustainability has been an emerging and growing topic in sports research in recent decades, there is a gap in the literature related to its relationship with other variables of great interest in sports administration such as quality, satisfaction, emotions and the future intentions of participants. According to Calabuig et al. (25), the integration of sustainable approaches in the internal processes of sports organisations improves aspects related to perceived quality, satisfaction and loyalty, while also reporting improvements in internal management related to the processes and key functions necessary to successfully manage an organisation (5). Therefore, we have put forward the following hypotheses:

H1a: Sustainability is a precursor to quality
H1b: Sustainability is a precursor to satisfaction
H1c: Sustainability is a precursor to positive emotions
H1d: Sustainability is a precursor to the intention to participate again.

### 2.2 Green practices in sporting events

Green practices are initiatives implemented by organisations to reduce the negative impact of their operation on the environment (26). These actions can be grouped into categories according to the scope of application, including waste management, education and communication, conservation of natural resources and wildlife, energy and water consumption, facilities management and transport, among others (27, 28).

The motivations of sports organisations to implement green practices stem from a variety of origins, in which concern for the environment is not the only factor. On occasions, they also respond to processes of organisational legitimacy, the need to set themselves apart from the competition and to adapt to current demands (3). In this regard, some authors, including Todaro et al. (5), suggest that to explain how sports organisations engage with the implementation of sustainable/green operational practices, we must know the various types of pressures to which they are subjected, including sports fans, the media, local governments, market expectations and society in general as producers of these demands. On the other hand, the main barriers or limitations that organisations face when it comes to implementing green practices in their operations are: the lack of appropriate financial and technological resources, the lack of awareness among the parties involved, as well as the lack of support and training for the implementation of green processes (27).

Regarding research related to the application of green practices, the literature notes that the main focus of interest has been on understanding consumer perception, green marketing strategies, the benefits of their application for organisations and the influence of these practices on participants' behaviour (29). Specifically in sport, there are studies that explore the implementation of green practices conceptually and empirically, focusing mainly on the motivations and limitations for their application (4, 30), chiefly in terms of sports spectators. However, although there are studies, it is important to note that much of the existing literature is based on sports in North America and Europe (5), without considering the situation elsewhere such as Central and South America. We only know of Chaves-Castro et al. (31) who have carried out a study in Costa Rica in which they linked green practices and the intention to participate again in the future, concluding that there was an indirect effect.

By implementing green practices in their management, organisations can obtain economic benefits, as operating costs can be reduced and the return on investment increased (32). In addition, they provide competitive advantages over those who do not implement green practices, while improving image, increasing good perceptions inside and outside the organisation, increasing fan identification with the organisation, while improving process efficiency and risk management (4, 5). In other words, the literature suggests that green practices can influence the perceived quality of the service provided, thus promoting a satisfactory experience of the event and the environmental care taken by the organisers (33), which could ultimately translate into a positive intention to participate again in the future (31). Therefore, we propose the following hypotheses:

H2a: Green practices are a precursor to quality.
H2b: Green practices are a precursor to satisfaction.
H2c: Green practices are a precursor to emotions.
H2d: Green practices are a precursor to the intention to participate again.

### 2.3 Emotions

Sport is made up of routine elements, of great perseverance, effort and dedication, but it is also an opportunity for relaxation, disconnection and often interaction with other people, which is why it is linked to emotional expression that creates intense experiences between those who practice it (34). Emotions are defined as a complex state of feelings in response to external or internal events of great importance to the organism (35), which instigate a specific response or behaviour (36). According to Bagozzi et al. (37), emotion is a state of preparation that arises from cognitive evaluations of events and can lead to specific actions. Emotion, therefore, is a complex psychological phenomenon that directs us towards behaviour in a coherent way and can influence decision-making (38). Hence, marketing studies frequently explore consumer emotions due to their impact on behaviour (39). Emotional reactions are precursors to general perceived image (40), satisfaction (41, 42), and behavioural intentions (43, 44).

Although emotions have been widely analysed in different consumption scenarios, their study in sport is relatively recent (45). Few studies include the role of emotions, although emotions and feelings are important factors for participation and development in sports services (46, 47). Regarding the role of emotions in the area of sports administration, some authors have integrated them as moderators in the model that relates quality, satisfaction and future intentions (14, 47, 48), starting from the point that a quality service generates satisfied participants, which will in turn feed into user loyalty and retention. Most studies that relate sports services and emotions focus on spectators as their target population (14, 46, 49), with few looking at users and/or athletes (47).

Therefore, due to the impact that emotions have on valuations and/or value judgements and on the future intentions and behaviours of sports service users (49, 50), it seems logical that emotions play an important role in the management of sporting events and that, if not taken into account, organisers would be missing a valuable opportunity to design events and meaningful experiences for athletes, reflected in satisfied participants willing to participate again in the future. Therefore, we have put forward the following hypotheses:

H3a: Emotions are a precursor to satisfaction
H3b: Emotions are a precursor to the intention to participate again.

### 2.4 Intention to participate again

Behavioural intentions are understood as the tendency of an individual to behave in accordance with their feelings, knowledge or evaluations of previous experiences (51), affecting, in the case of consumers, behaviours related to buying or using a service again (52). Biscaia et al. (49), looking specifically at the sports services market, understand behavioural intent as the participant's favourable intention to attend future games or recommend them to others, as well as to purchase products associated with the event. This intention is so powerful that although there is a time lapse between one's intention and the behaviour itself, it is possible that it will become behaviour, and the greater the intention, the more likely the behaviour (53).

A person's future intentions after participating in a sporting event are focused in two directions: firstly, the desire to participate in the event again in the future; and secondly, to recommend others to do so (51, 54, 55). Future intentions, according to studies, are directly related to perceived quality and satisfaction with the event or service, either to participate again or to recommend it to others (10, 11, 56). Similarly, studies have consistently shown that general satisfaction with the event or service may enhance the intention to increase physical activity afterwards (57) and participate in the following year's event (58–60). Therefore, following the different contributions of the authors cited, according to which there is a relationship between these constructs, we propose the following hypotheses:

H4a: Quality is a precursor to satisfaction.
H4b: Quality is a precursor to the intention to participate again.
H5: Satisfaction is a precursor to the intention to participate again.

**Figure 1:**
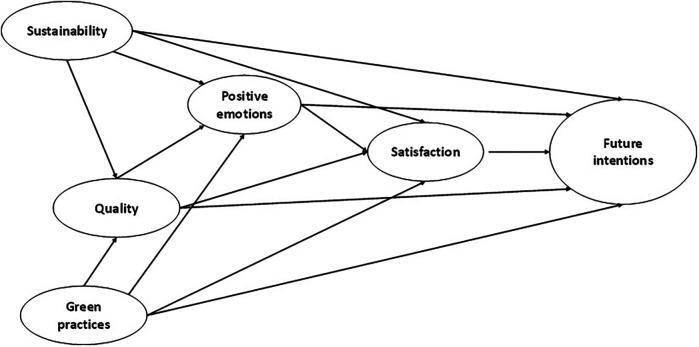
Model of relationships proposed between the study variables.

For all of the above reasons, we tested a model that relates both sustainability and green practices with quality, emotions, satisfaction and the intention to participate again in the future in a sporting event held in nature (Figure 1).

## 3 Methods

### 3.1 Data collection

We collected data through a self-administered questionnaire sent to 3,931 participants in recreational sports events held in nature in Costa Rica. A total of 781 responded satisfactorily, which means assuming a margin of error of 3.1% for a confidence level of 95%. All the participants were adults with an average age of 40.9 ± 11.9 years. 66.3% (518) were men and 33.7% (263) women. 62.3% (484) had a higher education. For 44.3% (346), this was the first time they had taken part in a sporting event held in nature, and the average number of times they had taken part in events of this type was 3.5.

### 3.2 Instruments

The instruments were answered using 7-point Likert scales. The first one analyses the perception that practitioners have in relation to sustainability (economic, social and cultural) through 9 items proposed by Lee and Jan (61). The second dimension, Green Practices, was measured using 6 items proposed by Morán-Gámez, Fernández-Martínez, Biscaia et al. (26). Quality was evaluated using 5 items adapted from the scale developed by Hightower et al. (62). Satisfaction was assessed using 5 items adapted from the scale developed by Hightower et al. (62). To measure positive emotions, 6 items were applied taken from Hosany and Gilbert (63). Finally, future intentions, specifically intentions to participate again, were measured using 3 items that Chaves-Castro et al. (31) used in their work. In addition, various sociodemographic items were added such as age, sex, education, and participation in sports events held in nature.

### 3.3 Data analysis

We used the IBM-SPSS and AMOS statistical packages (version 24.0) to perform the necessary analyses in this research. Firstly, we performed descriptive tests on the data (mean, standard deviation, asymmetry and kurtosis) and subsequently verified univariate and multivariate normality. We also carried out validity and reliability tests (correlation calculation, Cronbach's alpha, average variance extracted and composite reliability). We then carried out confirmatory factor analysis (CFA). We examined various indices to assess the model's goodness of fit: Chi-square (CMIN), Degrees of Freedom (DF), CMIN/DF ratio, Comparative Fix Index (CFI), Root Mean Square Error of Approximation (RMSEA), Tucker–Lewis Index (TLI), and Incremental Fit Index (IFI).

To determine univariate normality, values below 3 were considered normal for asymmetry and values below 10 for kurtosis (64). Multivariate normality was tested by means of Mardia's test (65). We verified validity and reliability using Cronbach's alpha, considering acceptable values ranging from 0.80 to 0.90 (66), Composite Reliability (CR), considering values above 0.6 to be adequate (67) and Average Variance Extracted (AVE), with values higher than 0.5 considered adequate (68). The indices to evaluate the goodness-of-fit of the model use as normative values: *χ*^2^/df < 5 (69), >0.90 for IFC, TLI and IFI, and <0.08 for RMSEA (68).

### 3.4 Procedure

The study was authorised by the Scientific Ethics Committee of the Universidad de Costa Rica. From there, we contacted the organisers of the events included in the study to inform them about the objectives of the project and the type of collaboration that was required of them. Once authorisation was obtained from the organisers, the questionnaire was sent to them so that they could email it to all participants once the event was over. In addition, we also sought the informed consent of all participants. It was done this way to guarantee the privacy of the participants' data and comply with Costa Rican Personal Data Protection Law No. 8,968, and thus, researchers never had access to the personal data of the competitors. Finally, each subject decided whether or not to fill out the questionnaire, since participation in the study was completely voluntary.

## 4 Results

Table 1 presents the values of asymmetry and kurtosis for the items used in the research. These values are below the expected limits, indicating that there is univariate normality. The value proposed by the AMOS.24 programme to measure multivariate normality was 811,992, lower than the value obtained using the *p* formula (*p* + 2) proposed by Mardia (65) to calculate multivariate normality which turned out to be 1,224, which demonstrates multivariate normality. Therefore the maximum likelihood method was used to calculate the model fit.

**Table 1:** Variables and items in the questionnaire applied .

Variable		Items	Mean	Standard Deviation	Asymmetry	Kurtosis
Quality		Overall, I received a high quality service at “The Event”.	5.66	1.55	−1.374	1.357
		Generally speaking, the service offered by the organisers of “The Event” at the venue was excellent.	5.80	1.58	−1.374	1.283
		The service offered during “The Event” was very good.	5.74	1.53	−1.326	1.178
		The service offered during “The Event” was excellent.	5.51	1.65	−1.184	.648
		The performance of the staff at “The Event” was excellent.	5.71	1.53	−1.302	1.153
Sustainability	Economic Sustainability	This event increases tax revenues in the local municipality.	5.80	1.56	−1.358	1.176
		This event promotes opportunities for local businesses.	6.27	1.31	−2.149	4.375
		This event attracts investment opportunities in the community where it was held.	5.75	1.62	−1.294	.935
	Cultural sustainability	This event facilitates participation in cultural activities.	5.86	1.57	−1.438	1.409
		This event facilitates the development of cultural activities.	5.88	1.52	−1.438	1.499
		This event contributes to the preservation of local culture.	5.81	1.60	−1.397	1.170
		This event has positive effects on the cultural identity of the community.	5.93	1.54	−1.516	1.540
	Environmental Sustainability	This event contributes to protecting the natural environment and wildlife habitats.	5.08	1.93	−.765	−.545
		This event contributes to protecting the biodiversity of the community.	5.06	1.89	−.735	−.511
Green practices		At this event, I was able to access biodegradable and/or recyclable products.	4.84	2.12	−.598	−1.004
		At this event, I was able to consume/access organic products.	4.83	2.10	−.585	−1.009
		The event organisers provided information on caring for the environment.	3.99	2.29	−.043	−1.494
		At this event, there were measures in place to efficiently use water and energy.	4.47	2.16	−.373	−1.239
		This event promoted the recycling of waste.	4.49	2.25	−.383	−1.331
		At this event, there were containers to collect the waste related to sports practice.	5.15	2.03	−.812	−.690
Satisfaction		I am happy with the experiences I had at this event.	4.25	1.13	−1.555	1.579
		I am satisfied with the experiences I had at this event.	5.70	1.75	−1.397	.996
		I truly enjoyed attending this event.	6.08	1.53	−1.874	2.752
		I was moved by the experiences I had at this event.	5.89	1.63	−1.601	1.784
		Attending as a competitor in this event was a pleasant experience.	6.06	1.55	−1.860	2.662
Positive Emotions		I had an enjoyable time competing in this event.	6.23	1.40	−2.224	4.547
		I was amazed after my participation in this event.	5.58	1.62	−1.179	.739
		I feel passionate about my performance at this event.	4.21	1.07	−1.385	1.341
		I feel a sense of inspiration regarding the event.	5.82	1.65	−1.498	1.397
		I have a feeling of joy about the event.	5.93	1.63	−1.744	2.316
		I have warm feelings about competing at this event.	5.57	1.84	−1.291	.614
	Intention to participate again	I feel a sense of pleasure after competing in this event.	6.02	1.78	−1,898	2.412
		I will recommend my friends and family attend this event in future editions.	6.09	1.62	−1.983	3.109
		If I had the opportunity to attend this event in future editions, I would repeat the experience.	6.16	1.63	−2.123	3.533

Below, Table 2 shows the correlation coefficients between the different constructs evaluated. We also carried out reliability tests (using Cronbach's alpha coefficients and CR) and validity tests (using AVE). Cronbach's alpha values were higher than.94, which indicates a high internal consistency of the items in the different constructs evaluated. The AVE presented values above.70, which indicates a high validity of the evaluated constructs. Finally, the CR values were above.95, suggesting a high reliability of the instrument used.

**Table 2:** Relationship between constructs, Cronbach's alpha, AVE and CR.

Variable	Mean ± SD	1	2	3	4	5	6	AVE	CR
1. Quality	5.68 ± 1.44	(.96)	.59**	.67**	.84**	.77**	.71**	0.86	0.97
2. Green practices	4.63 ± 1.89		(.94)	.75**	.54**	.54**	.43**	0.76	0.95
3. Sustainability	5.63 ± 1.36			(.94)	.67**	.62**	.56**	0.70	0.95
4. Satisfaction	5.60 ± 1.45				(.97)	.84**	.83**	0.91	0.98
5. Positive emotions	5.59 ± 1.40					(.95)	.80**	0.80	0.96
6. Participate again	6.08 ± 1.59						(.94)	0.90	0.96

SD, standard deviation; AVE, average variance extracted; CR, composite reliability; in parentheses Cronbach's alpha.

**p < .01.

Having performed the previous analyses and verified univariate and multivariate normality, we then tested the proposed model using the maximum likelihood method of estimation (ML). As we can see, the model fit indices show correct values for all the participating athletes (CMIN = 1107.025; DF = 498; CMIN/DF = 2.223; CFI = .951; RMSEA = .066; TLI = .945; IFI = .951).

Having verified the goodness-of-fit of the model, we calculated the direct effects of the model. The results showed that green practices are a precursor to quality, while sustainability influences quality, emotions and satisfaction. We also found that quality positively impacts satisfaction and emotions. Emotions for their part affect satisfaction and the intention to participate again. Finally, satisfaction is a direct precursor to the intention to participate again (Table 3).

**Table 3:** Analysis of direct effects.

Path			Standardised value	*p*
Quality	←	Green practices	.331	***
Quality	←	Sustainability	.543	***
Emotions	←	Sustainability	.233	.001
Emotions	←	Green practices	.021	.746
Emotions	←	Quality	.629	***
Satisfaction	←	Sustainability	.125	.017
Satisfaction	←	Quality	.412	***
Satisfaction	←	Emotions	.477	***
Satisfaction	←	Green practices	−.029	.520
Participate again	←	Satisfaction	.583	***
Participate again	←	Sustainability	.044	.443
Participate again	←	Sustainability	−.097	.189
Participate again	←	Emotions	.404	***
Participate again	←	Green practices	−.083	.097

*** *p* < .001.

Regarding the total effects, we noted that sustainability affects quality, satisfaction, emotions and the intention to participate again. Meanwhile, green practices also have a positive and significant relationship with quality, satisfaction, emotions and the intention to participate again. The model also shows that quality is a precursor to satisfaction, emotions and the intention to participate again. Emotions for their part are precursors to satisfaction and the intention to participate again. Finally, satisfaction precedes the intention to participate again (Table 4).

**Table 4:** Analysis of total effects.

Variables	Sustainability	Green practices	Quality	Emotions	Satisfaction
Quality	.543**	.331**			
Emotions	.574**	.229**	.629**		
Satisfaction	.623**	.216**	.712**	.477**	
Participate again	.587**	.103*	.572**	.683**	.583**

* *p* < .05.

** *p* = .001.

## 5 Discussion

Sports organisations are implementing strategies related to sustainability, due not only to concern for the environment, but also to the need to be competitive in the sports services market, since the adoption of such measures improves their image, helps to set them apart from the competition and meets current demands of various kinds (3). Therefore, the aim of this work was to explore the relationship between sustainability and green practices with the constructs quality, emotions, satisfaction and the intention to participate in future sporting events held in nature. The results have shown that sustainability affects quality, emotions, satisfaction and the intention to participate again. Green practices have effects on all variables related to value judgements and the intention to participate again, but with a lower effect than sustainability.

Having verified the validity and reliability of the instruments along with the model tested, we checked the different hypotheses. The results confirmed that sustainability is a direct and/or indirect precursor to quality (H1a), satisfaction (H1b) and positive emotions (H1c), results that can be described as congruent with the conclusions of some authors who mention how the inclusion of sustainable practices in sporting events has a favourable influence, not only on the image of the organisation, but also on participants' perceptions of the event (16, 70). In addition, the perception of sustainability can improve the experience of athletes during their practice by promoting a healthier and fairer environment, socially and economically speaking, generating a positive emotional response during the event (6). The incorporation of sustainable practices in sporting events conveys a positive message about the sustainable commitment of the organisation, which generates trust and respect among the participants, who connect and identify with the event, causing a greater degree of satisfaction (71).

With regard to the relationship between sustainability and the intention to participate again (H1d), the results do not confirm its direct effect. However, when observing the total effects, it is possible to establish a positive relationship between these variables and say that they have an indirect effect through both satisfaction and emotions, as shown in several studies (14, 60). Therefore, like Ferri et al. (72), we might assume that the contribution sustainability makes to the intention to participate again lies in the commitment that the event shows toward environmental conservation, the influence on sustainable social behaviour and the cost savings made thanks to these initiatives.

The results related to hypothesis 2 showed a positive relationship between green practices and quality (H2a). The existing literature supports this link because when participants positively value the implementation of environmentally friendly measures, they tend to better evaluate the event in terms of quality, since they perceive environmentally responsible organisations, favourably valuing the image and service delivery (4, 5). Recently, Morán-Gámez, Fernández-Martínez, Nuviala et al. (73) found that green practices and perceived quality have a positive and significant relationship within the context of sports services, specifically among athletes who use sports clubs, with women valuing this relationship to a greater extent. This result is of great relevance for sports organisations, as it demonstrates how the implementation of environmentally sustainable practices helps to improve the management and perception of sports services, whether it be an event or continuous service provision over time.

However, the results do not confirm green practices as a direct antecedent of satisfaction or emotions. Nevertheless, when analysing the total effects, it was possible to establish a positive relationship between green practices, satisfaction (H2b) and emotions (H2c), through perceived quality, verifying therefore these two hypotheses. Morán-Gámez, Fernández-Martínez, Nuviala et al. (73) found in their studies—one related to athletes belonging to sports clubs and the other to participants in a badminton sporting event—a significant and indirect relationship between green practices and satisfaction. The results lead to us suppose, like the aforementioned authors, that green practices alone are not enough to satisfy the needs of users and must be combined with other attributes to have an effect on this cognitive-affective dimension.

Similarly, emotions understood as a complex state of feelings in response to external or internal events that trigger behaviours (35, 36) lead us to study the effect that various stimuli, in this case green practices, have on emotions. The results have highlighted the indirect effect that green practices have on emotions in these athletes, differing from the findings of Quirante-Mañas et al. (74) in athletes who participated in an international senior badminton championship. At any rate, Calabuig et al. (48) and Cabello-Manrique, Nuviala et al. (14) had previously confirmed that emotions could be caused by the experience felt by sports spectators, to which, after this study, it can be added that ecological experiences, such as green practices, also affect the emotions of athletes. We might assume that the mere presence of green practices, regardless of any functional improvement they may bring to the service, would enhance an image associated with sustainability, which would favour the generation of affective responses to the service consumed (75), since emotions are the result of experiences caused by subjective personal reactions to different stimuli (74).

The results, as one might suppose, have shown a relationship, albeit indirect, between green practices and the intention to participate again, thereby confirming hypothesis H2d. In a similar study, Chaves-Castro et al. (31) found that green practices had an indirect effect on participants' future intentions, although the current study shows not only the mediating effect of quality and satisfaction, but also that of emotions. The effects of the variables quality and satisfaction on future intentions were higher than presented by green practices, a result that has been observed in this current research, where the effect of green practices was much lower than the rest of the variables included in the study.

Regarding hypothesis 3, emotions were confirmed as a precursor to satisfaction (H3a) and the intention to participate again (H3b). The results indicate that the positive emotions experienced by a participant in a sporting event are considered a relevant precursor to satisfaction. This is because positive emotions are closely linked to the way participants interpret the environment at the event. These emotions create an emotional and affective connection with the event, which in turn influences satisfaction and future intentions (14, 47, 48). So, if a participant experiences positive emotions, they are more likely to see the event as exciting, well organised, and rewarding, which contributes to a more positive perception of their experience. The results are consistent with the findings of Cece et al. (76) who assessed the importance of emotions in different processes that take place throughout a season in a competitive context, and Magaz-González et al. (47) and Quirante-Mañas et al. (74), who concluded the importance of emotions in the future intentions of participants in a European Duathlon Championship.

Additionally, the results indicate that quality is a direct antecedent of satisfaction (H4a) but not of the intention to participate again (H4b). However, the analysis of the total effects does show a significant relationship between the variables. These findings are consistent with previous studies (77–79), where the authors conclude that taking care of quality aspects in the development of sporting events is vital to participant satisfaction. That is, when participants feel that they have had a high-quality experience, where their expectations were fulfilled and their needs were met, they are more likely to feel satisfied with the event. In addition, the indirect relationship between quality and the intention to participate again is aligned with the findings reported in the literature, which note that quality is positively and significantly related to the future intentions of participants in sporting events (12, 72, 78).

Finally, the results of this study confirmed satisfaction as a precursor to the intention to participate again in the future. There is extensive evidence in the literature that satisfaction is a direct antecedent of future intentions in terms of attending future sporting events as spectators (14, 80) and the intention to participate again as athletes (10, 60, 77). Satisfaction is one of the main variables that predicts the behavioural intentions of users of sports services, whether they are clients of fitness centres (11), participants in a fun run (60), swimmers (10) or badminton players (74) and knowing the factors that determine this can act as a guide when designing sports services (73), since it has been shown that the greater the level of satisfaction, the greater the intention to participate again in the future (10, 60, 81). Knowing, therefore, which factors determine satisfaction, including aspects related to the organisation of the event, the provision of services, the complementary services available, the characteristics of the destination and satisfaction with the achievement (31, 81) is important, but also, based on this study, we must include green practices and sustainable practices as predictors of satisfaction.

The results of this study have revealed the positive effect that both perceived sustainability and green practices have on emotions, satisfaction and the intention to participate again in sports events held in nature. Similarly, perceived sustainability has a greater effect than green practices in all the variables analysed. Therefore, it is of greater interest for the organisers of such events to work on sustainability as a whole, since sustainability and green practices are interrelated and form a shared value (82, 83), than to limit themselves to proposing only green practices, since the results indicate that sustainability, which includes social, economic and environmental actions, has a greater effect on the rest of the variables, be they cognitive, affective, emotional or behavioural. We must not forget that working together on all three components that make up the TBL, in addition to providing internal benefits for the organisation, also yields external benefits for society (84). Thus, the organisers of a sporting event that implements measures in favour of sustainability can benefit from cost savings, contribute positively to the environmental problem of climate change and influence the behaviours and conduct of the participants. To conclude, when planning a sustainable sporting event, the organisers must offer alternatives in terms of accessibility and integration (e.g., enabling participation of functionally diverse people), the responsible use and consumption of natural resources (e.g., local, seasonal and/or organic), and respect positive environmental habits (e.g., recycling), which will have an impact on the three components of the organisation's TBL (21, 22, 84).

Despite the relevance of the results obtained in this research for the management of sports services, we must necessarily outline the series of limitations encountered and pose new research challenges. Firstly, the instrument used in this study was self-administered, which might be why not all participants completed the questionnaire. Although the sample is considered representative of the population studied, this could lead to a lack of important information. Therefore, the results should be interpreted with some caution because of these considerations. The target population of the study encompassed participants in various sports events held in nature, specifically in a single Latin American country, which may limit the generalisation of the results. Future research should aim to analyse the effect of sustainability on users of sports services in other environments and various types of activities so as to move beyond the limited scope of this study. Expansion of future research will help to confirm these results. The findings obtained pertain exclusively to users who have paid for a specific activity, without knowing the effect that such green practices have on users of different types of organisations, whether for profit or not. Further studies will therefore be needed to examine the relationship between sustainability and the intention to engage in physical activity in various types of organisations. Finally, this work is descriptive and does not allow us to assess the effects of such practices on athletes over time. Therefore, experimental research is required in the future to demonstrate that the sustainable management of sports services and activities provides favourable results for the organisation and for the athletes.

Finally, the results of this research show that sports organisations must offer high-quality, environmentally-friendly sports events that meet the demands of the market and which generate satisfactory experiences for athletes, which in the long run translate into repeat participation in the event. In addition, knowing and understanding the relationships between the variables studied allows for the implementation of strategies that contribute to improving the management of sporting events. Furthermore, this research is a starting point to generate other studies and analyse in greater depth the impact of implementing sustainability on the management of sporting events.

## Data Availability

The raw data supporting the conclusions of this article will be made available by the authors, without undue reservation.
